# Design and Initial Evaluation of a Low-Cost Microprocessor-Controlled Ankle Prosthesis

**DOI:** 10.3390/s26103257

**Published:** 2026-05-21

**Authors:** Zhanar Bigaliyeva, Abu-Alim Ayazbay, Sayat Akhmejanov, Nursultan Zhetenbayev, Aidos Sultan, Yerkebulan Nurgizat, Abu Jazar Ussam, Gulzhamal Tursunbayeva, Arman Uzbekbayev, Kassymbek Ozhikenov, Gani Sergazin, Yelubayeva Lazzat

**Affiliations:** 1 Department of Robotics and Technical Tools of Automation, Satbayev University, Almaty 050013, Kazakhstan; zh.bigaliyeva@satbayev.university (Z.B.); n.zhetenbaev@aues.kz (N.Z.);; 2 Department Aerospace and Electronic Engineering, Almaty University of Power Engineering and Telecommunications, Almaty 050013, Kazakhstan; a.sultan@aues.kz (A.S.);; 3 Department of Science and Innovations, Mukhametzhan Tynyshbayev ALT University, Almaty 050013, Kazakhstan; 4 Department of Prosthetic and Orthopedic Care, National Scientific Center for Development of Social Protection of the Ministry of Labor and Social Protection of the Republic of Kazakhstan, Almaty 050013, Kazakhstan

**Keywords:** ankle prosthesis, microprocessor control, ESP32 microcontroller, ball-screw actuation, IMU sensor

## Abstract

Lower-limb amputation remains a significant clinical and socio-economic challenge, while the high cost of microprocessor-controlled prostheses (MPKs) limits their widespread accessibility. This paper presents the design and preliminary laboratory-scale evaluation of a low-cost microprocessor-controlled ankle prosthesis intended as a feasibility-oriented alternative platform for future active prosthetic system development. Building upon the previously developed V1 mechanical architecture, an updated CAD model was created in the SolidWorks 2024 environment, and the kinematic configuration was refined using a ball-screw transmission (SFU1204-300) driven by a NEMA 17 stepper motor. The electronic control system integrates an ESP32 microcontroller, an MPU9250 inertial measurement unit (IMU), a limit switch for initial-position detection, and a WiFi-based REST API interface for communication and control. Laboratory no-load experiments demonstrated controlled positional behavior, repeatable angular response, and successful operation of the homing procedure within a motion range of 0–4200 motor steps. The prototype actively generated dorsiflexion–plantar flexion motion in the sagittal plane, while a passive inversion–eversion mechanism was incorporated and intended to improve structural adaptability. IMU-based measurements enabled preliminary monitoring of angular displacement and positional behavior during the experiments. The presented prototype represents an initial engineering feasibility study of a low-cost active ankle actuation architecture and provides a foundation for future investigations involving load-bearing experiments, biomechanical gait analysis, and closed-loop control implementation.

## 1. Introduction

Lower-limb amputation represents a complex medical and socio-economic problem that directly affects human mobility, work capacity, and overall quality of life. The development of modern prosthetic and orthopedic technologies has led to the emergence of various engineering solutions aimed at restoring the functional abilities of individuals with limb loss. Among these technologies, microprocessor-controlled prostheses (MPKs) are considered one of the most advanced and technologically sophisticated types of lower-limb prosthetic systems.

MPK systems are capable of adjusting joint motion in real time based on data obtained from multiple sensors. This control principle may improve gait stability, reduce the frequency of falls and balance loss, and enhance the flexibility and balance of walking patterns [1].

The clinical effectiveness of MPK systems has been investigated in several research directions. A number of studies have reported reductions in fall frequency and improvements in safety indicators compared with conventional non-microprocessor prostheses (NMPKs) [2,3]. Other investigations have reported a reduction in hip joint loading and a decrease in metabolic energy expenditure during walking [4,5]. In addition, improvements in gait stability, smoother movement patterns, and a reduction in the risk of secondary injuries have been documented [6,7,8,9].

Overall, the available literature indicates that MPK systems offer several functional, biomechanical, and safety-related advantages. However, some studies also report only moderate improvements or outcomes that vary depending on patient-specific factors. In addition, several studies emphasize that the clinical effectiveness of microprocessor-controlled prosthetic systems strongly depends on proper biomechanical tuning, patient-specific adaptation, and accurate experimental validation under dynamic loading conditions. Despite the promising functionality of modern MPK systems, many existing studies highlight the importance of evaluating torque characteristics, energy efficiency, gait biomechanics, and real-time control performance before broader clinical applicability can be considered.

Clinical investigations demonstrate that MPK systems can significantly improve the functional condition and quality of life of individuals with limb loss. Patients using MPKs typically show greater mobility, participate more actively in daily activities, and cope with physical loads more efficiently. From an economic perspective, although microprocessor-controlled prostheses involve a high initial cost, they may become cost-effective in the long term by reducing the incidence of secondary injuries and additional medical expenses, thereby increasing quality-adjusted life years.

At the same time, the technological complexity and high cost of advanced MPK systems limit their accessibility in many countries, particularly in regions with limited healthcare resources. This situation highlights the need to develop electronically controlled prosthetic systems that combine acceptable functional performance with lower production cost. Furthermore, many commercially available active prosthetic systems rely on complex actuator technologies and proprietary control architectures, which significantly increase manufacturing and maintenance costs. Therefore, the development of simplified and cost-effective electromechanical solutions remains an important engineering challenge, particularly for countries with limited access to advanced rehabilitation technologies.

### 1.1. Current Situation of Lower-Limb Amputation in Kazakhstan

In Kazakhstan, lower-limb amputation remains an important medical and social issue. According to official statistics, 3175 amputation cases were registered in the country in 2024, with the highest numbers recorded in the Karaganda, Turkistan, and Akmola regions as well as in the city of Almaty.

**Figure 1:**
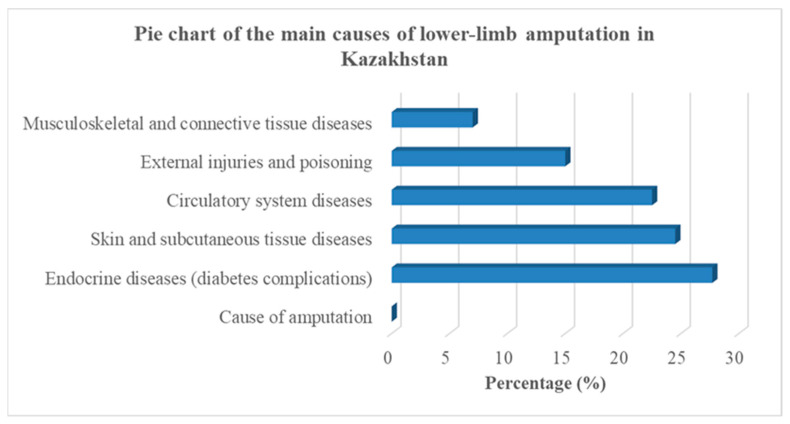
Distribution of the main causes of lower-limb amputation in Kazakhstan [10].

Analysis of the primary causes of amputation shows that the majority of cases are associated with chronic diseases. In particular, endocrine system disorders account for 27.7% of cases (mainly complications related to diabetes mellitus), diseases of the skin and subcutaneous tissues account for 24.5%, circulatory system diseases for 22.5%, external injuries and poisoning for 15%, and diseases of the musculoskeletal and connective tissues for 7%. The proportional distribution of the main causes of lower-limb amputation in Kazakhstan is illustrated in Figure 1.

The presented statistical distribution demonstrates the increasing demand for accessible lower-limb prosthetic rehabilitation systems in Kazakhstan, particularly for patients affected by diabetes-related complications and vascular diseases.

In addition, more than 95.3 thousand people in the country are classified as requiring prosthetic and orthopedic assistance. The correct selection of the amputation level directly influences the functional outcome of subsequent prosthetic rehabilitation. Lower-limb amputations are generally classified according to anatomical level as transtibial, transfemoral, and Syme amputations. According to Clinical Pub data, transtibial (below-knee) amputation is functionally more favorable because the knee joint is preserved, allowing more efficient gait biomechanics and reduced energy expenditure.

**Figure 2:**
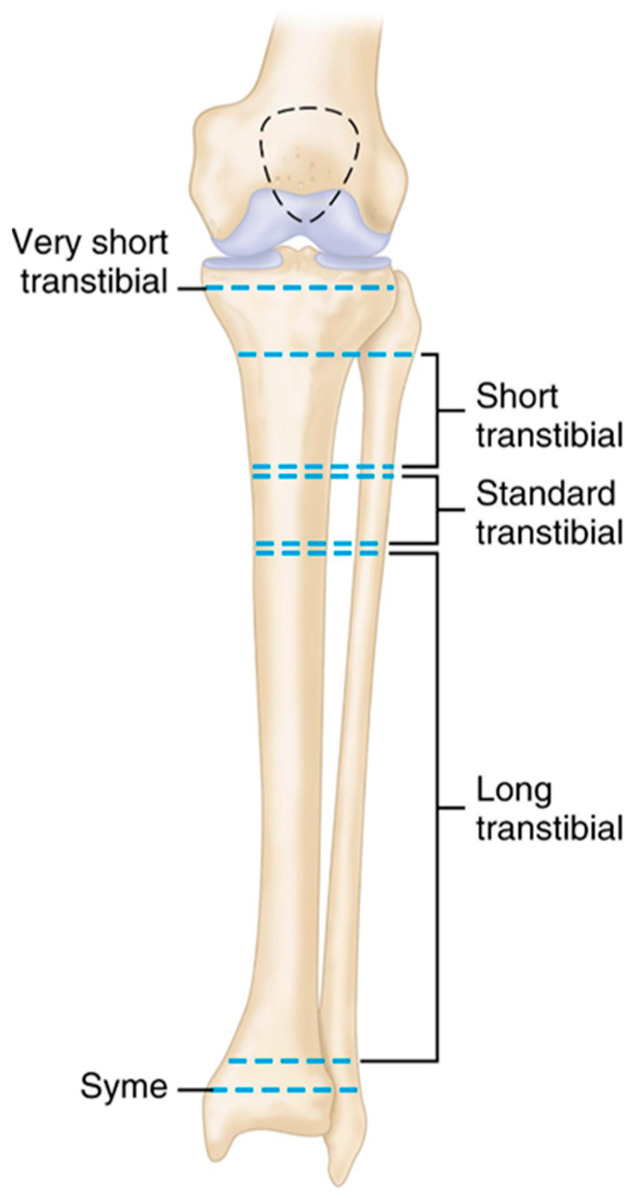
Classification of below-knee amputation levels of the lower limb [11].

Transtibial amputations are further classified based on the length of the residual limb as very short, short, standard, and long (Figure 2). Maintaining a sufficient residual limb length may improve prosthetic stability and controllability.

**Figure 3:**
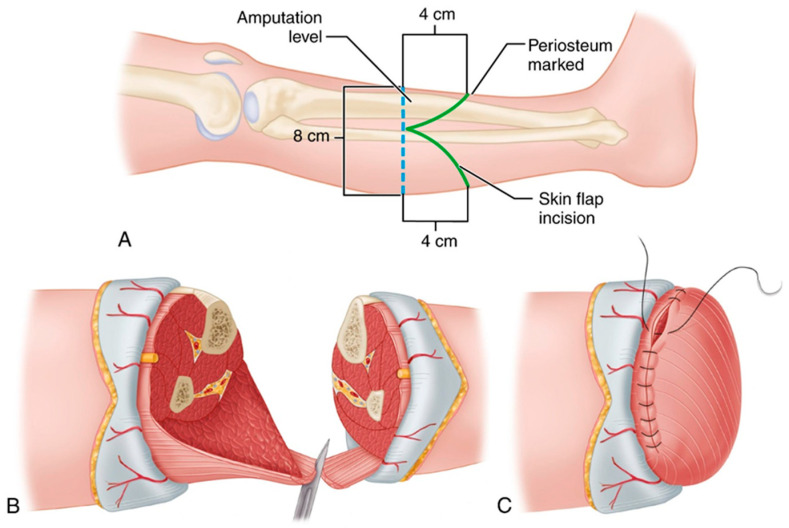
Surgical stages of transtibial amputation [11]. (**A**) Marking of the amputation level and skin flap incision; (**B**) Formation of muscle and soft-tissue flaps; (**C**) Final closure and suturing of the residual limb.

During the surgical stage, preservation of soft-tissue blood supply, proper formation of skin-muscle flaps, and adequate shaping of the bone ends are critical factors. These surgical stages are illustrated in Figure 3. Techniques such as myodesis and myoplasty may help reduce postoperative discomfort and facilitate subsequent prosthetic adaptation.

### 1.2. Current State of Prosthetic Manufacturing and Orthopedic Organizations in Kazakhstan

The prosthetic and orthopedic care system in Kazakhstan is coordinated by the Ministry of Labour and Social Protection of the Population of the Republic of Kazakhstan. According to data for 2025, approximately 82.8 thousand people (86.9%) are provided with technical rehabilitation devices. Each year, more than 70 thousand prosthetic and orthopedic products are supplied nationwide [12].

However, the majority of prosthetic assistance is still limited to passive mechanical prostheses. Active microprocessor-controlled prostheses are not produced serially within the country. Most modern systems currently used in Kazakhstan are manufactured in Europe and the United States and are typically supplied through import or refurbishment processes.

Although the installation of active MPK systems is technically feasible, their total cost—including installation and service—can reach eight to ten times the original price in the manufacturing countries. As a result, such technologies remain economically inaccessible for the majority of patients.

In recent years, an innovation center based on Ottobock technologies was opened in Astana. Nevertheless, at present it mainly operates at the level of assembly and service support. Overall, the prosthetic and orthopedic sector in Kazakhstan remains highly dependent on imported technologies, while domestic microprocessor-based production is still in an early stage of development [13].

Consequently, the limited accessibility of active microprocessor-controlled prostheses, dependence on imported technologies, and high system cost remain unresolved scientific and engineering challenges in Kazakhstan. In this context, the development of affordable domestically produced prosthetic systems is not only a technological objective but also an important socio-economic and rehabilitation-related priority. Low-cost active prosthetic devices may improve accessibility to modern rehabilitation technologies and reduce long-term dependence on imported systems.

The present study proposes an improved prosthetic design and microprocessor control architecture based on the mechanical and kinematic solutions previously developed in the earlier V1 prototype [14]. While the V1 version mainly focused on kinematic modeling and CAD analysis, the current work strengthens the mechanical structure, fully integrates a ball-screw actuation system, and introduces an electronic control module based on the ESP32 microcontroller. Compared with the previous prototype, the current system also aims to improve positional controllability and mechanical stability through the integration of a ball-screw transmission mechanism, which was selected with the aim of reducing backlash and improving repeatability of ankle joint motion.

In addition, angular displacement measurements and motion parameter recording are performed in real time using an MPU9250 inertial measurement unit (IMU), enabling preliminary experimental evaluation of the system.

The paper describes the structural architecture of the prosthesis, the control algorithm, the homing procedure, the REST API interface designed for remote control, and the sensor-based positional control mechanism. All experimental results were processed using angular data obtained from the IMU sensor, allowing preliminary evaluation of the relationship between motor step count and ankle joint rotation angle. A stepper motor was selected in the present study due to its low cost, simple control architecture, and ability to provide accurate incremental positioning without requiring complex feedback hardware. Although stepper-motor-based systems may demonstrate lower dynamic responsiveness compared with high-end BLDC or servo-driven prostheses, the proposed approach aims to provide a technically accessible and economically feasible solution for preliminary active prosthetic development.

At the current stage, the proposed system should be considered as a laboratory-scale engineering prototype intended for preliminary feasibility validation. Broader biomechanical and clinical applicability, and load-bearing behavior require further investigation through biomechanical testing and future user-centered studies.

The proposed solution is intended as a preliminary low-cost active prosthetic platform for future development of domestically adapted prosthetic systems in Kazakhstan. The present work primarily focuses on the mechanical integration, sensor-based positional control, and preliminary experimental validation of the proposed system under controlled laboratory conditions. The obtained results are intended to establish the technical feasibility of the prototype and provide a foundation for future biomechanical and clinical investigations.

## 2. Materials and Methods

### 2.1. Development of the Updated CAD Model

The updated prosthesis design was developed based on the mechanical and kinematic framework of the previous V1 prototype [14]. In the V1 model, a parametric 3D model created in the SolidWorks environment (Dassault Systèmes, Vélizy-Villacoublay, France) was presented together with a kinematic scheme and motion simulation. That version described the basic lever mechanism of the prosthesis, the location of the joint axis, and the general principle of force transmission.

**Figure 4:**
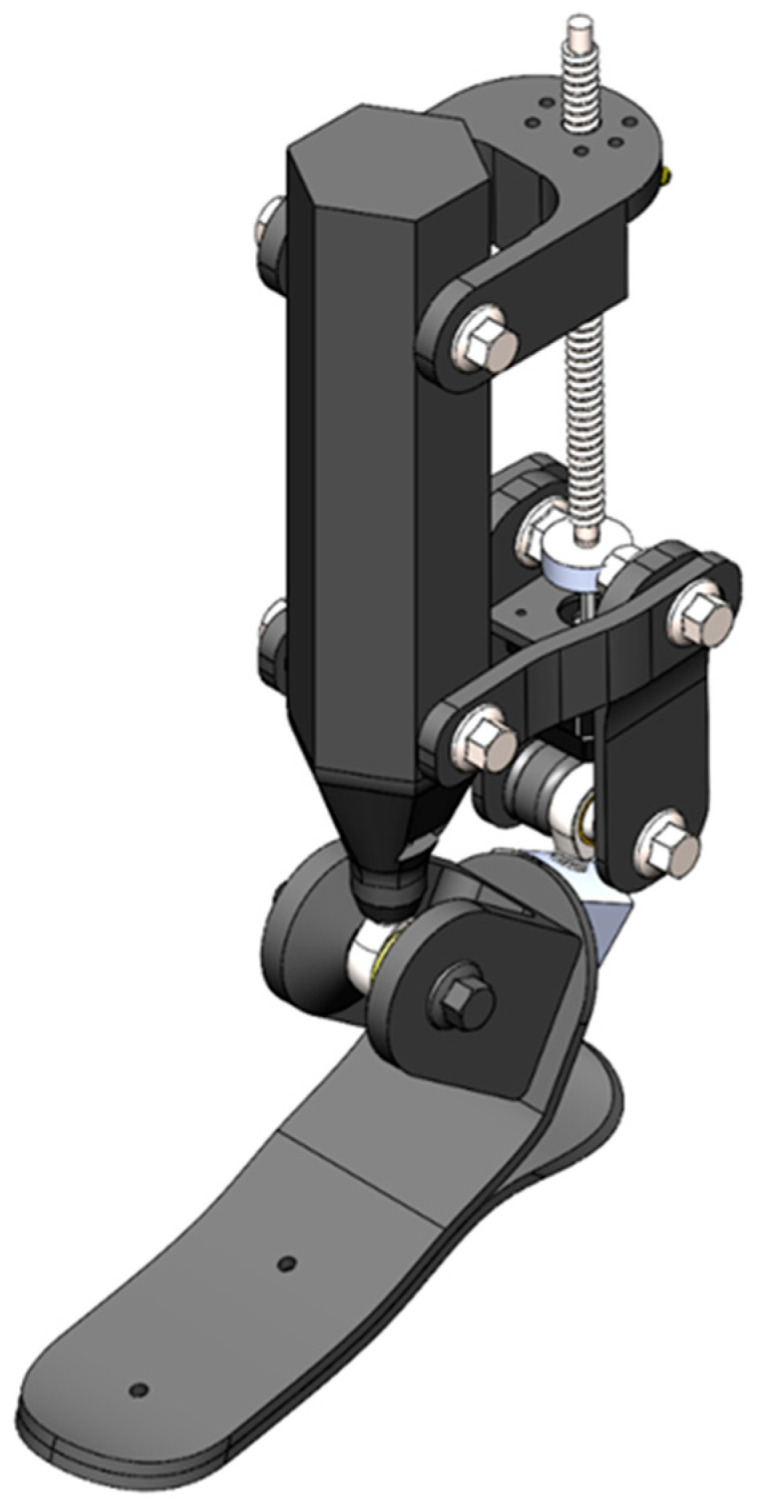
Complete CAD model of the updated active ankle prosthesis (general view).

In the present study, an improved version (V2) was developed in which the structural design was enhanced, the force transmission chain was redesigned, and the actuation system was reconfigured using a ball-screw mechanism. The overall structure of the updated CAD model is shown in Figure 4.

**Figure 5:**
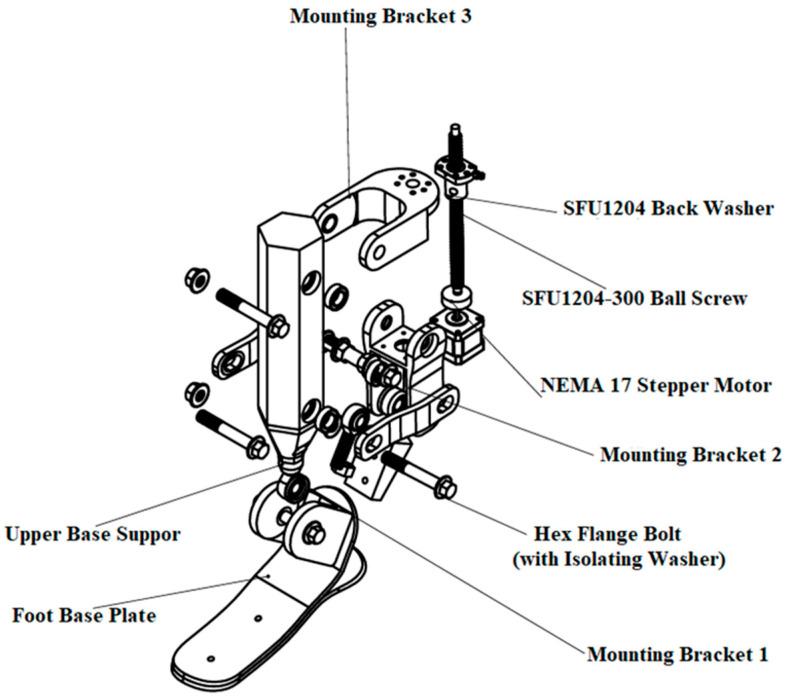
Component structure of the updated prosthesis and identification of the main elements.

While the V1 version employed a lever mechanism based on a simplified geometric configuration, the updated model uses a ball-screw transmission to convert linear displacement into rotational motion. This solution was intended to improve motion accuracy, reduce mechanical backlash, and support repeatable controlled motion. The ball-screw mechanism was additionally selected due to its relatively simple mechanical implementation, compact structure, and ability to provide precise incremental displacement under controlled actuation conditions. Compared with conventional linkage-based systems, the proposed transmission mechanism aims to improve positional controllability and facilitate integration with microprocessor-based control algorithms. The component composition of the assembly and the fastening elements are illustrated in Figure 5.

Figure 5 presents the principal structural elements of the updated prosthesis mechanism. In particular, the actuation system includes an SFU1204-300 ball screw and an SFU1204 back washer, which provide linear motion and maintain axial stability. A NEMA 17 stepper motor (17HS15-1704S) is used as the motion source. Structural fixation nodes are implemented through Mounting Bracket 1, Mounting Bracket 2, and Mounting Bracket 3, while the connections are realized using hex flange bolts with isolating washers. The lower support element is represented by the Foot Base Plate, while the upper supporting component is designated as the Upper Base Support.

A stepper motor was selected due to its low cost, availability, simple control architecture, and ability to provide relatively accurate discrete angular positioning without requiring complex encoder-based feedback systems. Although stepper motors may provide lower dynamic responsiveness compared with BLDC or servo-driven systems commonly used in commercial active prostheses, the present study focuses on the development of a preliminary low-cost engineering platform intended for feasibility validation and future optimization.

The proposed design follows a modular principle in which each structural unit is designed as an individual parametric component and subsequently integrated into a complete assembly model. This approach facilitates assembly and maintenance procedures and allows for future component replacement if required. The modular architecture was also selected to support future experimental modifications, including actuator replacement, sensor integration, and biomechanical optimization during subsequent prototype iterations.

During the CAD model development process, the following stages were carried out:

1. Kinematic recalculation—the lengths of the lever elements and the location of the rotation axis were revised and refined, and the force transmission trajectory was refined.
2. Parametric 3D modeling—all components were modeled individually in the SolidWorks environment, after which the complete assembly configuration was created.
3. Motion Study simulation—the joint rotation angle, displacement amplitude, and limit positions were preliminarily evaluated.
4. Force direction analysis—the geometric distribution of vertical loads acting during the stance phase was evaluated.
5. Assembly stability assessment—the positions of bolt connections and mounting brackets were refined from the perspective of mechanical rigidity.

During the simulation stage, the primary objective was to evaluate the geometric feasibility and controlled motion behavior of the mechanism rather than to reproduce full physiological gait loading conditions. Therefore, the present simulations should be interpreted as preliminary mechanical verification of the proposed architecture.

As a result, the updated design shown in Figure 5 incorporates several structural modifications compared with the V1 version, including a reinforced support architecture, a more precisely aligned force transmission system, and a mechanical base prepared for controlled actuation. The updated configuration is also intended to improve repeatability of the rotational trajectory and reduce undesired mechanical play during controlled movement generation.

In the updated model, the actuation system is aligned along the axial direction and the geometry of the force transmission mechanism is symmetrized. These design solutions aim to reduce bending moments generated during the stance phase and support increased structural stiffness of the mechanism. In addition, the foot section is connected through a hinged joint, allowing approximation of dorsiflexion and plantarflexion motion.

At the current stage of development, the mechanical architecture is intended primarily for laboratory-scale validation under controlled experimental conditions. Dynamic gait loading, long-term cyclic testing, and clinical biomechanical evaluation remain subjects this future investigation.

Thus, while preserving the fundamental kinematic concept of the V1 prototype, the updated CAD model focuses on improving mechanical reliability and manufacturability. This architecture also provides the structural foundation required for integrating a microprocessor-based control system and implementing a low-cost active prosthesis concept. The proposed design should therefore be considered as a preliminary engineering prototype intended for feasibility evaluation of low-cost sensor-controlled actuation mechanisms for transtibial prosthetic applications.

### 2.2. Structural Design of the Prototype

**Figure 6:**
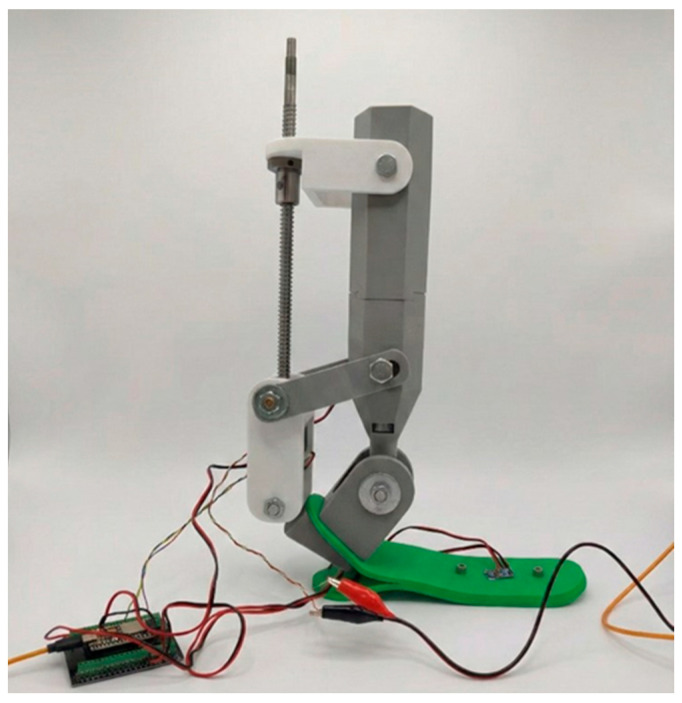
Physical prototype of a two-degree-of-freedom ankle mechanism with ball-screw actuation.

The laboratory prototype of the proposed ankle mechanism is shown in Figure 6. The structure consists of a vertically oriented actuation block, an intermediate lever mechanism, and a foot module incorporating the ankle joint.

As illustrated in Figure 6, the upper section contains a vertically oriented ball-screw transmission driven by a NEMA 17 stepper motor. The rotational motion of the motor is converted into linear displacement through the screw mechanism and transmitted to the lower ankle joint via a white intermediate lever. This mechanism actively generates dorsiflexion–plantarflexion motion in the sagittal plane. The selected transmission configuration allows relatively accurate incremental motion generation and simplifies the implementation of position-based control algorithms. In addition, the axial arrangement of the ball-screw mechanism is intended to improve force transmission alignment and may reduce undesired lateral displacement during operation.

The ankle joint itself is implemented through a primary rotational axis located within the gray lower block shown in the figure. The foot platform (green element) is attached to this axis and performs the main rotational movement. In addition, the design incorporates a passive hinge that allows limited inversion–eversion motion in the frontal plane, with an approximate range of ±8–10°. This passive degree of freedom is not motorized and was introduced to improve the structural adaptability of the mechanism. Although the inversion–eversion mechanism was not quantitatively evaluated in the present study, its inclusion was intended to provide limited frontal-plane compliance during future dynamic investigations.

The electronic control components, including the ESP32 microcontroller, motor driver, and power supply, are located separately from the mechanical prototype. This configuration allows safe and flexible testing of the system during the initial experimental stage. The external arrangement of the electronic modules additionally simplified access to the control architecture during debugging, sensor calibration, and preliminary motion testing procedures.

The entire structure was evaluated while fixed to a support base and was not tested under clinical load conditions. Therefore, the present experiments should be interpreted as laboratory-scale feasibility tests intended to preliminarily evaluate the operation of the actuation mechanism and sensor-controlled positioning system rather than to reproduce full physiological gait conditions.

No human subjects, amputee participants, or biomechanical gait simulators were involved during the present stage of experimental validation. Furthermore, dynamic load-bearing behavior, cyclic fatigue characteristics, and long-term mechanical durability were outside the scope of the current prototype investigation and remain subjects for future research.

Thus, the prototype shown in Figure 6 does not represent a full load-bearing prosthesis but rather an experimental actuation and control platform designed to investigate the drive system and sensor integration of a two-degree-of-freedom ankle mechanism. The developed structure should therefore be considered as a preliminary engineering prototype aimed at evaluating the feasibility of low-cost microprocessor-controlled ankle actuation prior to advanced biomechanical and clinical testing.

### 2.3. Electronic Control System

To support the functional capabilities of the updated prosthesis, a microprocessor-based control architecture was developed. The electronic system is designed to provide controlled motion generation, position monitoring, remote command reception, and transmission of telemetry data. The primary objective of the developed electronic architecture was to establish a stable and low-cost experimental control platform suitable for preliminary laboratory-scale validation of the active ankle mechanism.

**Figure 7:**
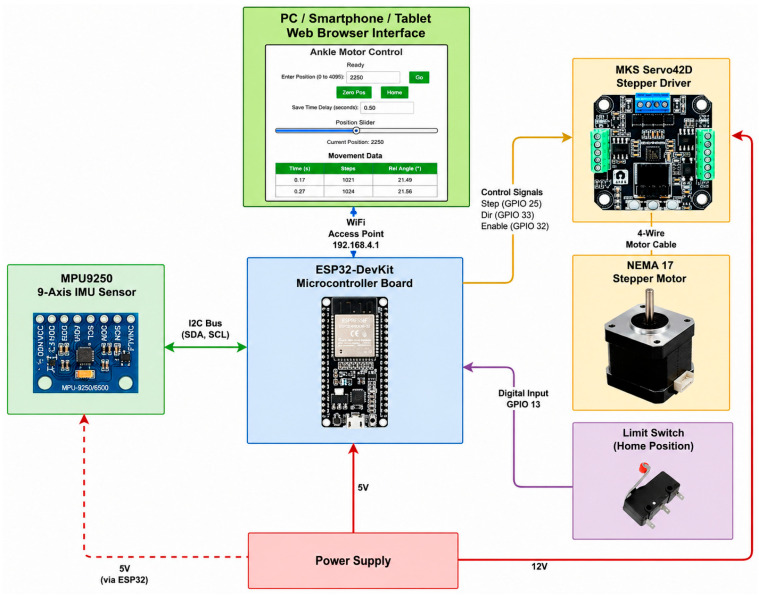
Hardware architecture of the microprocessor-based control system for the ankle prosthesis (ESP32-based structural diagram).

The structural diagram of the electronic architecture is presented in Figure 7. The ESP32-DevKit microcontroller (Espressif Systems, Shanghai, China) was selected as the central element of the system. This board integrates a WiFi module, sufficient GPIO resources, and adequate computational performance. Motion control is implemented using a NEMA 17 stepper motor together with an MKS Servo42D stepper driver. The motor driver receives control signals from the ESP32 (Step, Dir, Enable). As shown in Figure 7, these control signals are assigned to GPIO 25 (Step), GPIO 33 (Dir), and GPIO 32 (Enable).

The ESP32 platform was selected due to its low cost, integrated wireless communication capabilities, and sufficient computational performance for real-time position-control tasks. In addition, the selected architecture facilitates future integration of sensor-feedback and adaptive control algorithms.

To measure the joint rotation angle and spatial orientation, the system integrates an MPU9250 nine-axis IMU sensor. The sensor communicates with the ESP32 through the I2C bus (SDA, SCL). This configuration supports monitoring of angular displacement in real time and may provide a basis for implementing closed-loop control algorithms in future developments. At the current stage, the IMU sensor was primarily used for angular displacement acquisition and preliminary positional monitoring under controlled laboratory conditions.

For safety purposes and for detecting the initial mechanical position, a limit switch (Home Position) was incorporated into the system. This component is connected as a digital input through GPIO 13 and sends a signal to the control system when the mechanical limit position is reached. The homing procedure additionally reduces cumulative positioning errors and supports repeatability of the reference coordinate initialization process.

The power supply architecture is organized at two levels: a 12 V supply for the motor driver and a stabilized 5 V supply for the microcontroller and sensors. Such a separated power configuration helps reduce the influence of electromagnetic interference generated by high-current loads on the control electronics. This separation was introduced to improve electrical stability during experimental testing and to reduce sensor signal disturbances caused by motor switching operations.

**Figure 8:**
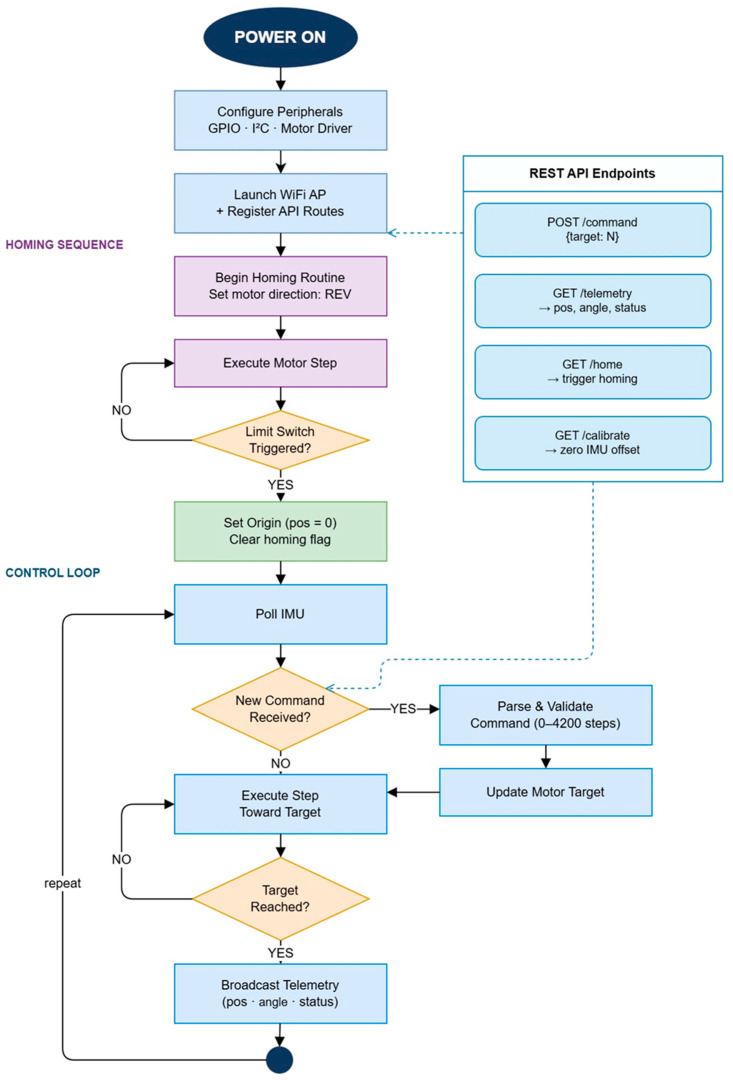
Block diagram of the prosthesis electronic control algorithm and REST API logic.

The software logic of the system is illustrated as a block diagram in Figure 8. After power-up (Power On), the peripheral modules (GPIO, I2C, and motor driver) are configured, after which the WiFi Access Point mode is activated and the REST API routes are registered. This interface allows the user to control the prosthesis motion via a web browser using a computer, smartphone, or tablet. The web-based architecture was selected to simplify experimental interaction with the prototype and to enable flexible real-time parameter monitoring without requiring specialized external software.

At the initial stage, a homing sequence is executed. The motor rotates in the reverse direction until the limit switch is triggered. Once the limit switch is activated, the position is reset to a zero reference (pos = 0), thereby defining the initial coordinate system. This step is essential for reducing the possibility of mechanical positioning errors and supporting repeatable initialization of the reference coordinate system.

The main control loop includes continuous acquisition of data from the IMU sensor. When a new command is received, the system interprets it within the range of 0–4200 motor steps, updates the target position, and drives the motor in the appropriate direction. Once the target position is reached, telemetry data (position, angle, and system status) are transmitted to the user via the web interface. The implemented control logic focuses primarily on positional control feasibility and experimental motion repeatability rather than on full biomechanical gait synchronization or adaptive locomotion control.

The REST API structure includes the following main commands:

− POST/command—set the target position.
− GET/telemetry—obtain position, angle, and system status.
− GET/home—move to the home position.
− GET/calibrate—calibrate the IMU zero offset.

The proposed software structure additionally supports future implementation of closed-loop feedback control, sensor fusion algorithms, and adaptive gait-related control strategies. However, these advanced control approaches were not implemented within the scope of the current preliminary prototype investigation.

The proposed electronic control system follows an open architecture principle and supports modular expansion. This approach provides a framework for future implementation of sensor-based feedback algorithms, adaptive control strategies, and closed-loop stabilization systems. At the current stage, the developed control system should therefore be interpreted as a laboratory-scale engineering platform intended for preliminary validation of microprocessor-controlled ankle actuation and sensor integration concepts.

### 2.4. Experimental Test Setup

**Figure 9:**
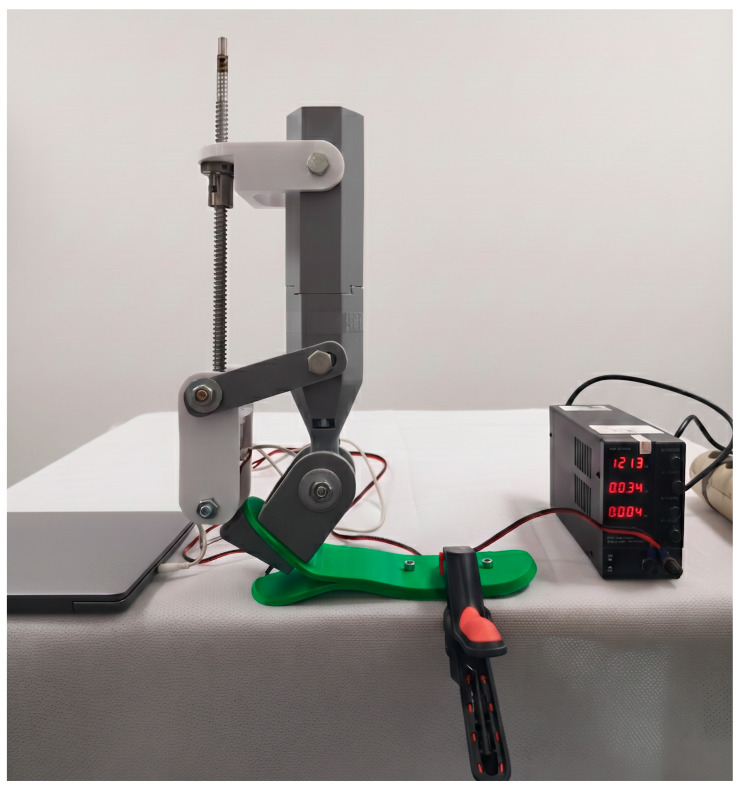
Laboratory experimental setup of the two-degree-of-freedom ankle mechanism: stationary fixation, external power supply, and control electronics.

The overall configuration of the laboratory experimental setup is shown in Figure 9. The prototype was tested in a stationary configuration, fixed to the surface of a laboratory table. This arrangement provided mechanical stability and allowed the drive and control characteristics of the system to be evaluated at an initial stage without dynamic or load-related influences. The stationary fixation configuration was intentionally selected to facilitate evaluation of the fundamental actuation behavior of the mechanism and to minimize the influence of uncontrolled external variables during preliminary validation experiments.

The setup includes the ankle mechanism prototype, a stepper motor driver, an ESP32-based control system, and a stabilized external laboratory power supply. As can be seen in Figure 9, the power supply unit is positioned separately from the prototype and provided regulated power during the experiments. Locating the control electronics and the power unit outside the mechanical structure allows flexible system configuration and supported controlled laboratory testing conditions.

During the experiments, the ankle mechanism was evaluated under no-load conditions. The purpose of this stage was to examine the generation of dorsiflexion–plantarflexion motion in the sagittal plane, to determine the relationship between motor steps and angular displacement, and to assess the operational stability of the control algorithm. The experiments were therefore focused primarily on preliminarily evaluating the feasibility of the actuation and positional control architecture rather than reproducing full biomechanical gait conditions or physiological load-bearing behavior.

The passive degree of freedom in the frontal plane (inversion–eversion) is not motorized and was therefore not quantitatively investigated in these experiments. Although the inversion–eversion mechanism was mechanically implemented to provide limited frontal-plane compliance, its biomechanical influence and quantitative motion characteristics remain subjects for future investigation.

Before each experiment, the mechanical zero position was established using a limit switch. Motion parameters such as speed and acceleration were preset to conservative values in order to support stable and low-impact operation of the actuation system. This approach was chosen to support the initial functional validation of the prototype. Conservative motion parameters were additionally selected to reduce undesired vibration, minimize mechanical shock loading, and support repeatability of the experimental measurements.

The joint angle was monitored using the MPU9250 inertial measurement unit (IMU). The IMU sensor was mounted on the foot platform and used to record angular variations in the sagittal plane. Measurement results were displayed in real time, and the motor step counts were compared with the corresponding measured angular values. At the current stage, the IMU-based measurements were intended primarily for preliminary angular monitoring and preliminary experimental evaluation of positional repeatability under laboratory conditions.

No human participants, amputee subjects, or biomechanical gait simulators were involved in the present experiments. Furthermore, torque generation, energy efficiency, cyclic loading behavior, and long-term durability characteristics were not evaluated within the scope of the current laboratory validation stage.

Thus, the experimental setup shown in Figure 9 does not represent a full load-bearing or clinical test environment. Instead, it serves as a laboratory platform designed to evaluate the actuation and control architecture of the two-degree-of-freedom ankle mechanism under controlled experimental conditions. The obtained results should therefore be interpreted as preliminary feasibility data intended to support future biomechanical, load-bearing, and clinical investigations of the proposed prosthetic system.

## 3. Experimental Study

### 3.1. Experimental Procedure

During the experimental study, the main functional motions of the ankle mechanism dorsiflexion (upward lifting of the forefoot) and plantar flexion (downward movement of the forefoot) were investigated. All experimental data were obtained specifically from these two motion trajectories. The tests were conducted under stationary, no-load conditions and were intended to preliminarily evaluate positional-control behavior of the actuation system. At the current stage, the experiments were focused primarily on preliminary feasibility validation of the actuation mechanism and sensor-based positional control architecture rather than on reproducing complete physiological gait conditions.

**Figure 10:**
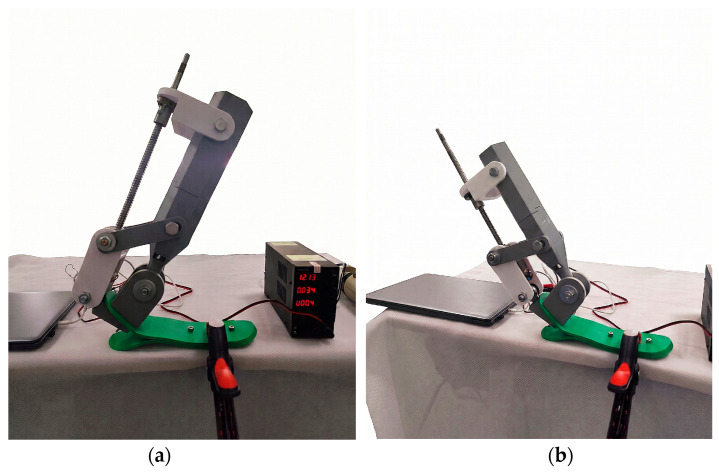
Experimental configurations of the ankle prosthesis: (**a**) dorsiflexion motion; (**b**) plantar flexion motion.

Figure 10a,b present the experimental configuration of the prosthesis mechanism in the dorsiflexion and plantar flexion states. In Figure 10a (left), the configuration illustrates the dorsiflexion phase, in which the lever mechanism rotates upward through the ball-screw actuation system, lifting the front portion of the foot. The configuration shown in Figure 10b represents the plantar flexion motion, where the actuation system operates in the opposite direction, causing the foot platform to tilt downward. The motion was generated by control impulses (step counts) supplied to the NEMA 17 stepper motor.

During the experiment, the relationship between the number of motor steps and the angular displacement of the ankle joint was recorded. Each movement was executed up to its limiting positions, and the rotation angle, motion consistency, and repeatability were preliminarily evaluated. The system performed the motion smoothly and without abrupt transitions, suggesting reduced mechanical backlash during controlled motion and acceptable alignment of the kinematic pairs. The experiments therefore focused on evaluating motion repeatability and controlled positional behavior under laboratory conditions rather than on assessing full biomechanical performance characteristics such as torque generation or gait-loading response.

Thus, the dorsiflexion and plantar flexion movements shown in Figure 10a,b represent the primary operational modes of the prosthesis and served as the basis for determining the experimental relationship between control impulses and angular displacement.

**Figure 11:**
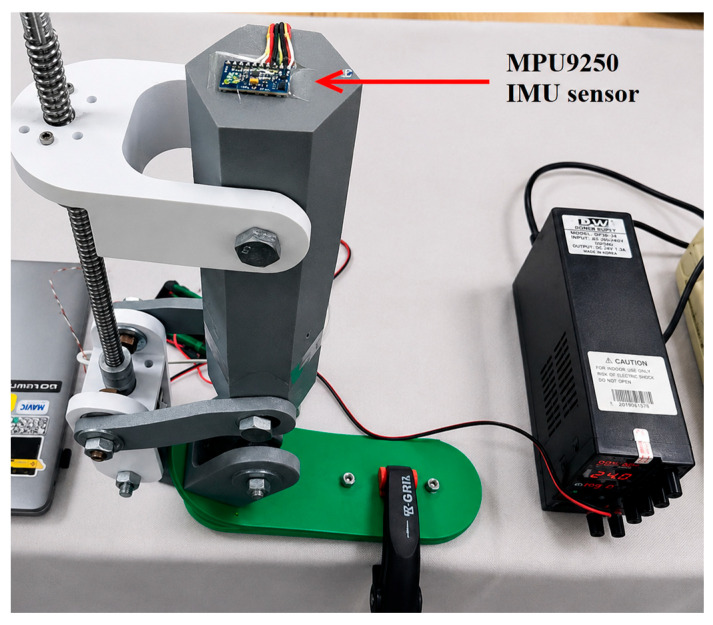
Location of the MPU9250 IMU sensor mounted on the prosthesis during the experiments.

During the experimental investigation, all angular displacement values and motion-related dynamic data were obtained using the MPU9250 IMU sensor. As shown in Figure 11, the sensor was mounted on the upper housing of the prosthesis and recorded the real-time spatial orientation of the mechanism together with the rotation angle of the ankle joint. By combining accelerometer, gyroscope, and magnetometer data, the IMU supported high-frequency monitoring of angular variations during dorsiflexion and plantar flexion motions. Experimental angular analyses were based on measurements obtained from this sensor.

At the present stage, the IMU measurements were used primarily for preliminary angular monitoring and comparative positional analysis. The obtained sensor data were not validated against external gold-standard biomechanical measurement systems such as optical motion-capture systems or force platforms, which represents one of the limitations of the current study.

**Figure 12:**
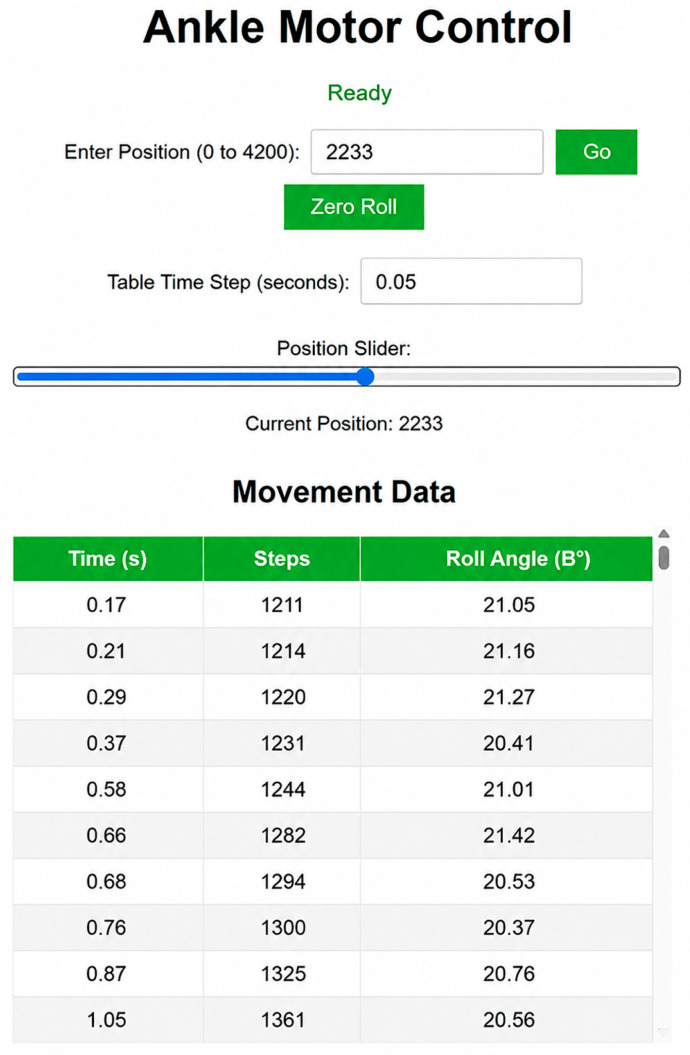
Web-based interface for controlling the ankle mechanism and real-time recording of motion data.

Figure 12 presents the web-based control interface used during the experiments. Through the “Ankle Motor Control” panel, the operator can specify the target position of the ankle joint within the 0–4200 step range. The “Zero Roll” button is used to set the zero angular offset according to the IMU sensor. In addition, motion control was performed using the Table Time Step parameter and a positional slider interface.

Before each experiment, the mechanical zero position was determined using a limit switch. After this initialization step, the system operated in a positional control mode according to the predefined number of motor steps. Motion parameters, including speed and acceleration, were configured with conservative values in order to support stable and low-impact movement during the initial validation phase of the prototype. Conservative control parameters were additionally selected to reduce vibration effects, minimize abrupt mechanical transitions, and support repeatability of the experimental measurements.

As shown in the “Movement Data” table in Figure 12, the parameters Time, Steps, and Roll Angle were recorded in real time during each experiment.

The collected data supported preliminary analysis of the relationship between motor step commands and the measured angular values. At this stage, the system was evaluated without dynamic loading, focusing exclusively on positional control accuracy and motion repeatability. Torque generation, power consumption, cyclic fatigue behavior, energy efficiency, and long-term mechanical durability were outside the scope of the current experimental investigation and remain subjects for future research.

Furthermore, no human participants, amputee users, or biomechanical gait simulators were involved during the present experimental stage. Consequently, the obtained results should not yet be interpreted as clinical-performance indicators but rather as preliminary laboratory-scale feasibility data for validating the proposed actuation and control architecture.

Therefore, the conducted experiments represent a laboratory-level validation aimed at preliminarily demonstrating the capability of the two-degree-of-freedom ankle mechanism to generate active sagittal-plane motion and demonstrating the operational feasibility of the integrated sensor feedback system. The obtained experimental results establish a preliminary engineering foundation for future load-bearing, biomechanical, and clinical investigations of the proposed low-cost ankle prosthesis system.

### 3.2. Evaluation of Prototype Performance

The operational performance of the prototype was evaluated by analyzing motion consistency and repeatability of sagittal-plane motion generation. The evaluation criteria included the temporal variation in motor steps, the angular displacement of the ankle joint, and the correspondence between these parameters. During the experiments, motion was performed within the 2700–1200 step range, which represents the positional control window of the motor and corresponds to the investigated sagittal angular displacement. The experiments were conducted under controlled laboratory conditions and were intended primarily for preliminary evaluation of positional controllability and motion repeatability rather than for full biomechanical performance assessment.

**Figure 13:**
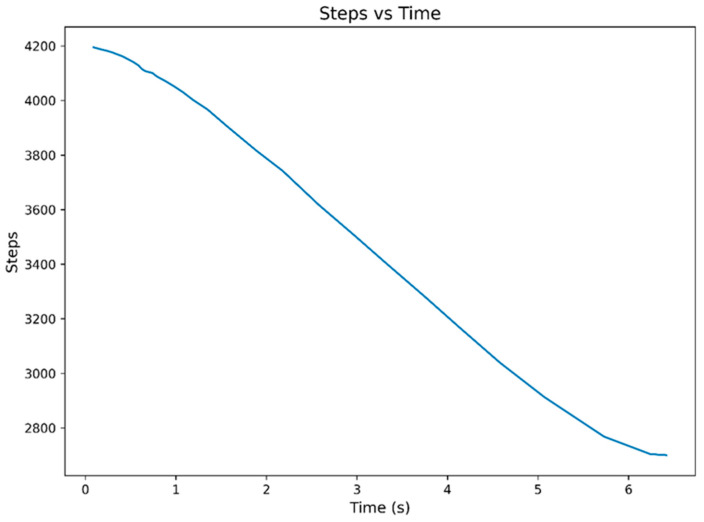
Temporal variation in the stepper motor position (Steps vs. Time).

Figure 13 illustrates the dependence of motor steps on time, showing that the motor position changes smoothly and monotonically. This indicates controlled operation of the drive in positional control mode and suggests the absence of significant abrupt fluctuations in the mechanical system. The continuous character of the curve additionally demonstrates stable transmission of the control impulses and consistent operation of the ball-screw actuation mechanism during the experimental procedure.

**Figure 14:**
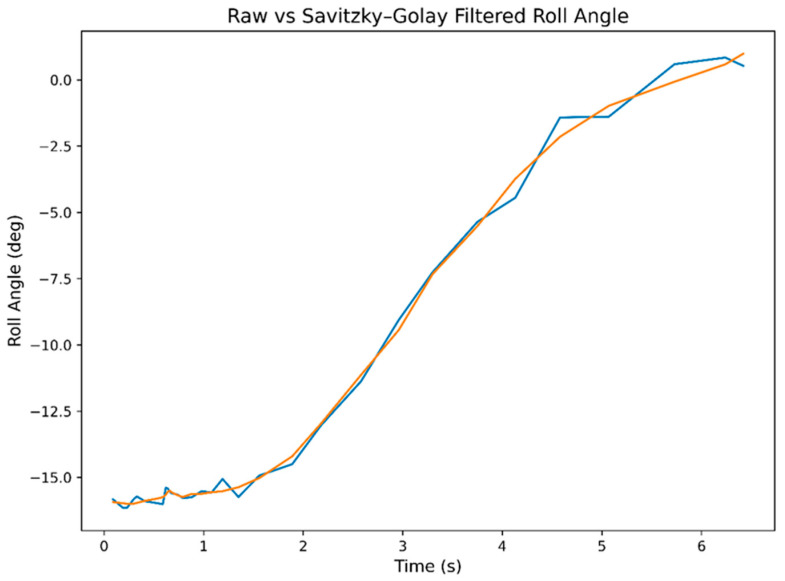
Temporal variation in the ankle joint angle: raw IMU signal and Savitzky–Golay filtered curve.

Figure 14 presents the time evolution of the ankle joint angle. The blue curve represents the raw IMU signal, while the orange curve shows the signal processed using a Savitzky–Golay filter (window length = 11, polynomial order = 3). The filtering procedure reduced high-frequency noise while preserving the primary dynamic profile of the motion. The results indicate a consistent functional relationship between changes in motor steps and the measured angular response. Minor fluctuations observed in the raw IMU signal can be attributed to sensor noise and small mechanical clearances within the prototype structure. The Savitzky–Golay filtering procedure improved visualization of the principal kinematic trend without significantly distorting the measured angular behavior.

During the motion, a gradual angular change was recorded from approximately −16° to about +1°. The observed small phase delay can be explained by the elasticity of the mechanical lever system and minor structural mismatches. Considering that the experiments were conducted under no-load laboratory conditions, the obtained results indicate controlled positional operation of the proposed stepper-motor-driven actuation system. The experimental data indicate preliminary positional repeatability and stable control behavior within the investigated motion range under laboratory-scale validation conditions.

**Figure 15:**
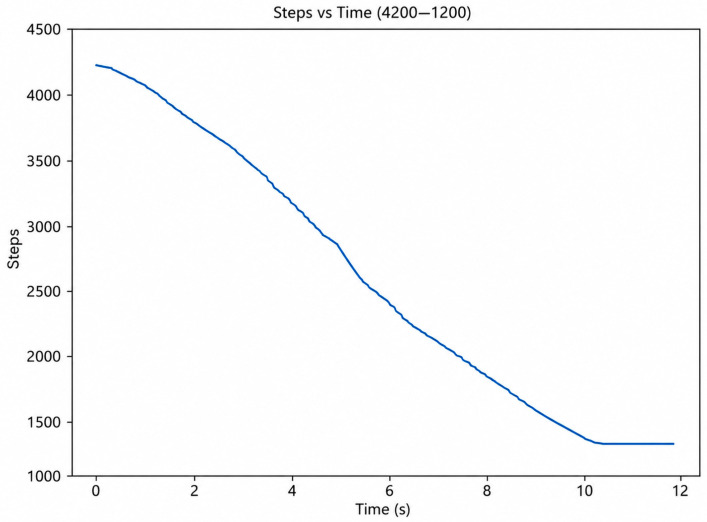
Temporal variation in the stepper motor position (Steps vs. Time), 4200–1200 range.

To further evaluate the operational capability of the prototype, additional experiments were performed within the full 4200–1200 step range. Figure 15 illustrates the temporal variation in the motor step position during the complete motion cycle. The step count decreases monotonically and smoothly from 4200 to 1200, indicating stable control signal delivery and continuous operation of the drive system without abrupt transitions. Stabilization near the final position indicates repeatable execution of the commanded trajectory and demonstrates the repeatability of the positional control architecture.

**Figure 16:**
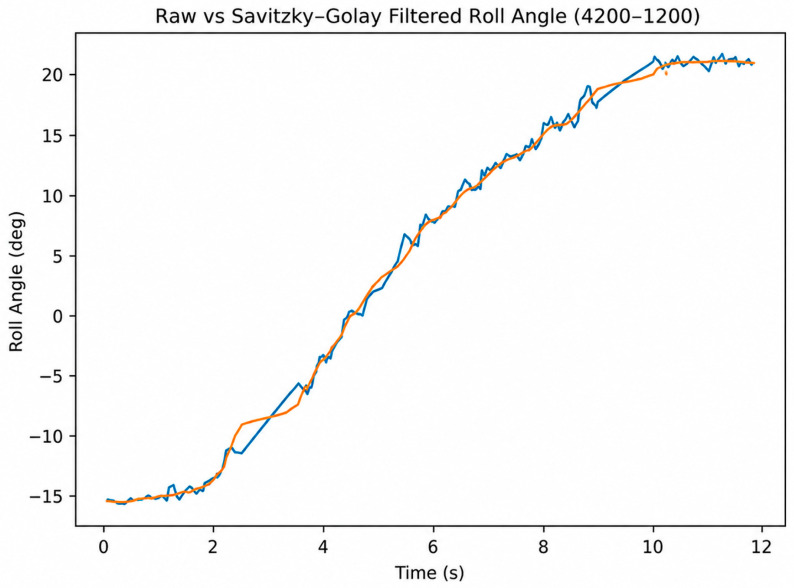
Temporal variation in the ankle joint angle (raw and Savitzky–Golay filtered data), 4200–1200 range.

Figure 16 presents the corresponding temporal variation in the ankle joint angle measured by the IMU sensor. The angle gradually increases from approximately −16° to about +21°, corresponding to the complete sagittal-plane motion cycle of the mechanism. The filtered curve clearly reveals the primary kinematic behavior of the system while suppressing high-frequency oscillations present in the raw signal. A consistent functional relationship between motor step variation and angular displacement is observed throughout the experiment, suggesting consistent synchronization between the mechanical and electronic subsystems of the prototype.

Overall, the obtained experimental results demonstrate that the proposed prototype is capable of generating controlled sagittal-plane motion under laboratory conditions. A consistent functional relationship was observed between motor step count and ankle joint angle, while the filtered IMU data highlighted the principal dynamic characteristics of the movement. At the current stage, the experiments should be interpreted as preliminary feasibility validation of the actuation and sensor-feedback architecture rather than as full clinical or biomechanical evaluation of prosthetic performance.

Furthermore, parameters such as torque generation, power consumption, cyclic fatigue behavior, dynamic gait loading, and long-term durability were not investigated within the scope of the present study and remain subjects for future research. No human participants or biomechanical gait simulators were involved during the experimental validation stage.

The obtained results nevertheless establish a preliminary engineering foundation for future development of low-cost microprocessor-controlled ankle prostheses and support the feasibility of integrating sensor-based positional control into the proposed actuation system.

### 3.3. Preliminary Analysis of the Obtained Results

As a result of the conducted experiments, the kinematic and control characteristics of the active ankle actuation module were preliminarily evaluated under laboratory no-load conditions. The experimental analysis focused on the relationship between motor step variation and ankle joint angular displacement during sagittal-plane motion. To improve clarity and reduce redundancy, only representative full-range experimental results are presented in the current version of the manuscript.

The graphs presented in Figure 13, Figure 14, Figure 15 and Figure 16 indicate a consistent functional relationship between the stepper motor position and the ankle joint angle:

− The motor step variation exhibited a monotonic character;
− The angular displacement remained continuous and without significant abrupt transitions;
− Stabilization was observed near the terminal positions;
− The Savitzky–Golay filtering procedure reduced high-frequency oscillations while preserving the principal kinematic trend of the motion.

Based on the experimental observations, the prototype exhibited an angular displacement range of approximately 35–38° during the complete sagittal-plane motion cycle. The obtained results additionally indicate a consistent correspondence between motor step count and measured angular displacement throughout the investigated operating range.

Minor deviations observed in the raw IMU measurements are attributed to sensor noise, small structural clearances, and elastic effects within the mechanical linkage system. Nevertheless, the filtered data demonstrate preliminary positional repeatability and stable motion behavior under controlled laboratory conditions.

The obtained experimental results indicate that:

1. The proposed stepper-motor-driven actuation system is capable of generating controlled sagittal-plane motion;
2. A consistent relationship exists between motor step variation and ankle joint angular displacement;
3. The integrated IMU sensor system enables real-time monitoring of angular behavior;
4. The proposed control architecture provides controlled positional operation during both forward and reverse motion directions.

At the current stage, the presented results should be interpreted as preliminary feasibility validation of the mechanical actuation and sensor-feedback architecture rather than as a full biomechanical or clinical assessment of prosthetic performance. Parameters such as torque generation, energy efficiency, cyclic loading behavior, and long-term durability were not investigated within the scope of the present study.

The performed analysis indicates that the prototype demonstrates controlled positional behavior and repeatable motion characteristics under laboratory-scale experimental conditions. The obtained findings establish an initial engineering foundation for future load-bearing experiments, biomechanical gait analysis, and future biomechanical and user-centered prototype investigations.

## 4. Discussion

The experimental results demonstrated controlled and repeatable kinematic behavior of the proposed active ankle prosthesis under laboratory conditions. A consistent functional relationship between motor step count and angular displacement was observed, demonstrating consistent transmission behavior and controlled positional operation. The obtained results indicate that the proposed actuation architecture is capable of generating controlled sagittal-plane motion using a low-cost stepper-motor-driven mechanism integrated with sensor-based positional feedback.

The developed prototype incorporates two mechanical degrees of freedom. The primary degree of freedom corresponds to active dorsiflexion–plantar flexion motion in the sagittal plane and is generated through the ball-screw actuation system driven by the stepper motor. The second degree of freedom corresponds to inversion–eversion motion in the frontal plane and is implemented as a passive mechanical hinge. Thus, only the dorsiflexion–plantar flexion motion was actively controlled and quantitatively investigated during the present experimental study, while the passive inversion–eversion mechanism was included primarily to improve structural adaptability and potential lateral compliance during future gait-related investigations.

The total sagittal-plane angular range reached approximately 39–40°, including ≈22.05° dorsiflexion and ≈−17.9° plantar flexion. According to biomechanical literature, normal level-ground walking requires approximately 10–20° dorsiflexion and 15–25° plantar flexion. Therefore, the achieved motion range falls within the angular ranges commonly reported for basic gait-related ankle motion. However, activities such as stair climbing and high-dynamic movements require further investigation of torque generation, dynamic response characteristics, and load-bearing performance. The present results should therefore be interpreted as preliminary laboratory-scale kinematic feasibility evaluation rather than confirmation of full clinical gait capability.

Quantitative evaluation of control performance was conducted to complement the previously qualitative description. The measured steady-state positional deviation was within ±0.3–0.5°, with repeatability in the same range. Angular resolution was approximately 0.012–0.015° per motor step, and homing accuracy was ±1 step. Step response time for full-range motion was approximately 10–11 s. These parameters are summarized in Table 1.

Although the obtained positional metrics demonstrate controlled positional behavior under controlled laboratory conditions, the current validation does not yet include torque characterization, cyclic fatigue evaluation, energy-consumption analysis, or biomechanical assessment under physiological loading. Consequently, the reported parameters should be interpreted within the context of preliminary no-load feasibility testing.

It should be emphasized that all validation experiments were conducted under stationary no-load laboratory conditions. Consequently, load-bearing capacity, structural rigidity under physiological loading, torque output, power generation, dynamic response under cyclic gait conditions, and positional holding capability under external forces remain to be evaluated. These aspects will be addressed in future investigations through mechanical strength analysis, load-based testing, and gait simulation experiments. Furthermore, no human participants, amputee users, or biomechanical gait simulators were involved during the current stage of validation.

To provide a comparative overview of hardware cost, a detailed Bill of Materials (BOM) analysis was conducted. The primary hardware components and their costs are presented in Table 2.

Total prototype hardware cost: ≈265 USD. A comparative evaluation with commercially available microprocessor-controlled prosthetic systems is presented in Table 3.

The comparison highlights a considerable cost difference while acknowledging differences in validation level, dynamic capability, and clinical maturity. The proposed prototype is therefore not intended to directly compete with clinically validated commercial prosthetic systems but rather to demonstrate the feasibility of a significantly lower-cost experimental platform for active ankle actuation research.

From an actuation perspective, most contemporary active ankle prostheses utilize BLDC or servo motors due to their high torque density and dynamic response. In contrast, the present design adopts a stepper motor combined with a ball-screw transmission. This configuration was selected to emphasize positional controllability, mechanical simplicity, modularity, and cost efficiency. The trade-off includes limited dynamic speed and the need for further optimization under load. The selection of a stepper-motor-based architecture was motivated primarily by affordability, ease of implementation, and suitability for preliminary laboratory-scale prototyping.

The mechanical design incorporates a passive inversion–eversion degree of freedom (±8–10°), which may improve lateral adaptability on uneven terrain. Quantitative evaluation of this degree of freedom under loading conditions remains a topic for future research. At the current stage, the passive inversion–eversion mechanism was not included in the active control architecture and therefore was not quantitatively characterized during the present experiments.

Although no clinical trials or user-centered validation have yet been performed, the demonstrated positional stability and repeatability provide an engineering basis for future closed-loop control strategies, impedance regulation, and adaptive gait algorithms. Future investigations should additionally include biomechanical gait analysis, load-bearing experiments, dynamic-response characterization, and comparison with gold-standard motion-analysis systems.

Overall, the results establish laboratory-level engineering feasibility and provide a preliminary foundation for further biomechanical and clinical development. The presented findings should therefore be interpreted as an initial engineering validation of a low-cost two-degree-of-freedom ankle actuation concept rather than as a clinically validated prosthetic solution.

## 5. Conclusions

This study presented an updated mechanical and electronic architecture of a low-cost microprocessor-controlled ankle prosthesis. The project was developed with consideration of the clinical and social needs of Kazakhstan and aims to present a preliminary engineering platform for future development of microprocessor-controlled prosthetic systems.

The work preserves the kinematic concept introduced in the earlier V1 prototype while strengthening the mechanical structure and integrating the control system. The updated CAD model underwent parametric recalculation, the force transmission geometry was refined, and the assembly stability was enhanced. In addition, an ESP32-based electronic control system, an IMU sensor, a limit switch, and a REST API architecture with a web-based interface were implemented.

The proposed solution is based on a modular mechanical principle and an open software architecture, allowing further system development and extension. The developed prototype relies on low-cost and widely available components and represents an initial laboratory-scale engineering step toward the development of domestically accessible prosthetic technologies.

The conducted study led to the following engineering conclusions:

− The updated mechanical architecture of the prosthesis demonstrated controlled linear motion generation using a ball-screw actuation system (SFU1204-300) under laboratory no-load conditions.
− Positional control functionality was implemented through integration of a NEMA 17 stepper motor and an MKS Servo42D driver.
− The ESP32-based control system enabled remote WiFi-based operation and real-time telemetry transmission.
− A homing procedure based on a limit-switch mechanism provided repeatable initialization of the mechanical reference position.
− IMU-based angular monitoring enabled preliminary evaluation of the relationship between motor step variation and ankle joint displacement.
− The developed prototype supported preliminary feasibility evaluation of constructing a low-cost experimental active ankle platform using commercially available components.

At the present stage, the obtained results should be interpreted as a feasibility-oriented laboratory evaluation of the proposed actuation and control architecture rather than as a full biomechanical or clinically validated prosthetic system. The experiments were conducted under stationary no-load conditions and did not include physiological gait loading, torque characterization, cyclic durability analysis, or human-subject evaluation.

The developed prototype incorporates two mechanical degrees of freedom, including actively controlled dorsiflexion–plantar flexion motion and a passive inversion–eversion mechanism intended to provide limited frontal-plane compliance. However, only the active sagittal-plane motion was quantitatively investigated within the scope of the present study.

Future research will focus on the following directions:

1. Implementation of closed-loop control algorithms (e.g., PID or impedance control);
2. Integration of force sensors for load measurement;
3. Analysis of energy efficiency and optimization of battery autonomy;
4. Conducting biomechanical gait simulations and load-bearing experiments;
5. Experimental comparison with external motion-analysis systems;
6. Investigation of the passive inversion–eversion mechanism under loading conditions;
7. Development and optimization of future multi-degree-of-freedom prosthetic architectures.

Overall, the presented findings establish a preliminary engineering foundation for future development of low-cost sensor-controlled ankle prosthesis systems and support preliminary evaluation of integrating microprocessor-based positional control into a laboratory-scale active prosthetic platform.

## Figures and Tables

**Table 1:** Quantitative Control Performance Metrics of the Proposed Ankle Prosthesis.

Parameter	Value
Total sagittal angular range	≈39–40°
Maximum dorsiflexion	≈22.05°
Maximum plantar flexion	≈−17.9°
Steady-state positional error	±0.3–0.5°
Positional repeatability	±0.3–0.5°
Angular resolution	≈0.012–0.015°/step
Step response time	≈10–11 s
Homing accuracy	±1 step
Bandwidth	To be evaluated under load

**Table 2:** Bill of Materials (BOM) of the Proposed Active Ankle Prosthesis.

No.	Component	Description	Quantity	Cost (USD)
1	NEMA 17 Stepper Motor	Electromechanical actuator	1	25
2	Ball-Screw (SFU1204-300)	Linear transmission mechanism	1	45
3	MKS Servo42D Driver	Motor control module	1	35
4	ESP32 Microcontroller	Control and communication system	1	10
5	MPU9250 IMU Sensor	Angular displacement measurement	1	12

**Table 3:** Comparative Cost Analysis with Commercial Active Ankle Prostheses.

Device	Manufacturer	Type	Approximate Cost (USD)
Empower Ankle	Ottobock	Active ankle prosthesis	30,000–40,000
Meridium	Ottobock	Microprocessor foot	25,000–35,000
PowerFoot One	BionX Medical	Active prosthesis	20,000–30,000
Proposed Prototype	This study	Active ankle prosthesis	≈265

## Data Availability

The data presented in this study is available on request from the corresponding author.
